# The Effect of Mustard Gas on Salivary Trace Metals (Zn, Mn, Cu, Mg, Mo, Sr, Cd, Ca, Pb, Rb)

**DOI:** 10.1371/journal.pone.0126162

**Published:** 2015-05-12

**Authors:** Elham Zamani Pozveh, Ahmad Seif, Parichehr Ghalayani, Abbas Maleki, Ahmad Mottaghi

**Affiliations:** 1 Department of Oral Medicine, Dental School Faculty, Khorasgan (Isfahan) Branch, Islamic Azad University, Isfahan, Iran; 2 Department of Chemistry, Boroujerd Branch, Islamic Azad University, Boroujerd, Lorestan, Iran; 3 Department of Oral Medicine, Isfahan University of Medical Sciences, Isfahan, Iran; 4 Department of Oral and Maxillofacial Surgery, Dental School Faculty, Khorasgan (Isfahan) Branch, Islamic Azad University, Isfahan, Iran; CINVESTAV-IPN, MEXICO

## Abstract

We have determined and compared trace metals concentration in saliva taken from chemical warfare injures who were under the exposure of mustard gas and healthy subjects by means of inductively coupled plasma optical emission spectroscopy (ICP-OES) for the first time. The influence of preliminary operations on the accuracy of ICP-OES analysis, blood contamination, the number of restored teeth in the mouth, salivary flow rate, and daily variations in trace metals concentration in saliva were also considered. Unstimulated saliva was collected at 10:00–11:00 a.m. from 45 subjects in three equal groups. The first group was composed of 15 healthy subjects (group 1); the second group consisted of 15 subjects who, upon chemical warfare injuries, did not use Salbutamol spray, which they would have normally used on a regular basis (group 2); and the third group contained the same number of patients as the second group, but they had taken their regular medicine (Salbutamol spray; group 3). Our results showed that the concentration of Cu in saliva was significantly increased in the chemical warfare injures compared to healthy subjects, as follows: healthy subjects 15.3± 5.45(p.p.b.), patients (group 2) 45.77±13.65, and patients (Salbutamol spray; group 3) 29 ±8.51 (P <0.02). In contrast, zinc was significantly decreased in the patients, as follows: healthy subjects 37 ± 9.03(p.p.b.), patients (group 2) 12.2 ± 3.56, and patients (Salbutamol spray; group 3) 20.6 ±10.01 (P < 0.01). It is important to note that direct dilution of saliva samples with ultrapure nitric acid showed the optimum ICP-OES outputs.

## Introduction

Saliva is a dilute fluid located in mouth, secreted by the major salivary glands such as parotid, submandibular, and sublingual; and many minor salivary glands. Human saliva contains 99.5% water, while the other 0.5% consists of organic and inorganic components, trace elements, etc. [1]. A healthy person’s mean daily saliva production ranges from 1 to 1.5L [2]. Saliva is considered as one of the most necessary and critical dilute fluid used to preserve and maintain the health of oral tissues and as a source of non-invasive investigation of metabolism. However, it receives little notice until its quantity diminishes or its quality becomes altered [3–6]. The metals in saliva play several important roles such as taste perception, affecting activity of many enzymes, making and intensifying bone and teeth, hardening tooth enamel, neutralizing mouth environment, preventing tooth decay, etc. Therefore, it would be very important to measure metals concentration in saliva [7, 8].

Measurement and analysis of trace elementary (metals and non-metals) in saliva still have some complexities such as very low concentrations of analyses, extensively unreliable salivary compositions, the lack of a standardized analytical method, and the nonattendance of reliable reference values [9]. Although, there are several methods for measurement of trace metals concentrations, one of the most important methods is inductively coupled plasma optical emission spectroscopy (ICP-OES) because of its high accuracy and extreme sensitivity.

Sulfur mustards—Bis (2-chloroethyl) sulfide- were first used widely in World War I by the German army against the British and Canadian soldiers in Belgium in 1917. Since then, this gas was used many times in several wars particularly during the Iraq-Iran war (1980–1988). This vesicant gas was last used by the Iraqi Army in the Mersad mission in Ilam zone in 1988.

The sulfur mustards [(C_2_H_4_Cl)_2_S], usually known as mustards gas, are a class of related cytotoxic and vesicant chemical warfare agents that cause many acute and chronic diseases including respiratory, skin, and eye diseases [10].

The harmful effects of mustards gas on saliva have been studied by various research groups recently. For example, Zamani et al found that victims of chemical warfare had a high degree of tooth decay and low levels of saliva production as compared to average healthy subjects [11]. Yarmohammadi et al investigated the salivary levels of secretary IgA, C5a and alpha 1-antitrypsin in patients who were exposed to sulfur mustards 20 years earlier and found that their salivary IgA and alpha 1-antitrypsin levels were much higher than the control group even for those with a single episode of exposure [12].

The aim of the present investigation is to determine the difference between the salivary levels of trace metals of healthy subjects and those exposed to chemical warfare. In this research, the chemical warfare injures suffer from respiratory problems, low volume of saliva, and having more restored teeth comparing healthy subjects. Additionally, the study determines the blood contamination and daily variations in trace metal levels in saliva. Our study points out that the trace metals concentrations ratio is different in those injured in chemical warfare when compared to healthy subjects. Furthermore, to our knowledge, there hasn't been any related investigation in this area and this work is the first of its kind.

## Materials and Methods

### Subjects

An expert distributed the questionnaires among more than 300 chemical warfare's injured individuals (They were harmed in Mersad mission in Iraq-Iran war in Ilam zone) and two groups of fifteen-people were selected from them. In order to obtain reliable data, we choose two 15-people groups and all individuals within these groups had the same medical condition. Because of such medical criterion, our groups ended up to be relatively small. This is because of the fact that among the statistical population (N = 300), only there were 30 patients who were male, in the age range of our test, volunteer and had no other diseases. Moreover, from these 30 patients, some of them were not willing to stop their regular medicine; therefore, we had to divide them into two mustard gas exposed groups (group 2 and 3).

The first group or blank group—who went voluntary to the special clinic and are visited by the special doctors and then the healthy ones were selected according to the doctors' diagnose and finally have been selected randomly—consisted of 15 male healthy subjects (mean age: 45.74±2.11 years) who were non-smokers and had not taken any medicine within 15 days before the experiment. The second group consisted of 15 male injured in chemical warfare (mean age: 46.5±2.38 years) who were non-smokers and had not taken their regular medicine within one day before the experiment, the patients could not stop taking their medicine more than 24 hours. The third group included 15 male injured in chemical warfare (mean age: 46.16±1.78 years) who were non-smokers and had taken their regular medicine; the Salbutamol spray 2 times per day. Salbutamol spray is taken by the patients who have not severe respiratory problems and usually by taking 2 times per day, the problems were relatively resolved. Moreover, those who had severe respiratory problems and other diseases did not want to participate in the test. Furthermore, the exposure of mustard gas was unexpectedly and the time of exposure was 9±3 minutes and the time that the injures were under the exposure and then taken to the hospital was 30±5 minutes.

It is important to note that the 30 injured participants were suffering from respiratory diseases, and all the environmental conditions were almost identical for all of them. Furthermore, none of the participants was exposed to any environmental trace metals before the experiment; moreover, they had different numbers of treated teeth, but they almost had no carious teeth. Our research proposal was approved by The Ethics and Research Committee of Islamic Azad University, Khorasgan Branch and also Boroujerd Clinic of Special Patients (proposal number: 3132700) and all the participants provided written informed consent.

### Saliva collection

All saliva samples were taken between 10:00–11:00 am to reduce variability in salivary composition. All Participants were requested to avoid doing oral activities such as chewing, drinking and tooth brushing for at least 90 minutes before sample collection. The participants were asked to rinse their mouths for 2 minutes with 20 ml of ultrapure distilled water and immediately after the rinsing the subjects carefully spat their saliva into 15 ml cleaned low-density polyethylene (LDPE) bottles. This process was performed for roughly 10–20 minutes to get 6–14 ml of saliva per participant. To determine daily variations for healthy subjects (group 1) in salivary levels of trace metals, sample collection was taken during 7 days. Meanwhile, the sample collection for chemical warfare subjects (group 2) was taken during 7 days and for chemical warfare subjects (group 3) was taken 2 times per day for 1 day. After collection, the flow rate of saliva (ml min^-1^) and the number of restored teeth in the mouth were recorded. To measure the level of blood pollution, saliva samples were centrifuged immediately at 4000_˟_g for 20 min. the supernatants for each sample were sub-sampled to two aliquots of 100μl, and these aliquots were stored at -65°C until use. 70% of super pure nitric acid was added to the rest of the sample (0.05 ml of nitric acid per 1 ml of saliva sample). These samples were then stored at -25°C until ICP-OES analysis.

### Preliminary operations for ICP-OES study

To determine the pretreatment method that produced the most accurate ICP-OES results, we compared three pretreatment methods: direct dilution with perchloric acid and nitric acid, direct dilution with nitric acid and direct dilution with distilled water. A total number of 10 elements (Ca, Cu, Mg, Zn, Rb, Mo, Sr, Cd, Mn and Pb) in the saliva sample were determined for the comparison. Saliva samples of six healthy subjects (10 ml per person) were collected to obtain a 60 ml sample. Firstly, for samples that were diluted directly with HNO_3_ and HClO_4_, 1 ml aliquots were diluted with 70% HNO_3_ (9.5 cc) and 60% HClO_4_ (0.5cc). Two more aliquots of 1 ml were spiked with known metal quantities (5 and 10 μgl^-1^). Secondly, for samples that were diluted directly with nitric acid, 1 ml aliquots were diluted 10-fold with 70% super pure nitric acid. Two more aliquots of 1 ml were spiked with known metal quantities (5 and 10 μgl^-1^). Finally in the third level, samples were diluted directly with ultrapure distilled water instead of nitric acid, and the same procedure of the second level was repeated. To assess the daily variation in trace metal levels in saliva or to compare the levels of trace metals in saliva between healthy subjects and chemical warfare injured ones, samples were diluted directly with nitric acid, as this was determined to be the pretreatment operation of samples that yielded the optimum ICP-OES results.

### Determination of trace metal concentrations in saliva samples

The concentrations of trace metals (μgl^-1^) in saliva samples were measured by ICP-OES. At first, by the use of standard solution (1000 ppm stock solution), different concentrations were prepared for each metal and then the calibration curve was drawn. When R2 = 0.99 (Coefficient of Determination), the unknown metal concentrations of samples were injected into ICP-OES (Varian (735): Nebulizer V-groove). Each test sample was analyzed three times by the same method. The limit of detection (LOD) for each metal was defined as three times the standard deviation of the healthy subjects' samples.

### Measurement of blood contamination in saliva samples

To measure the amount of blood contamination in saliva samples, the concentration of transferrin in saliva samples was calculated using a salivary blood contamination enzyme immunoassay kit (Salimetrics, State College, PA, USA).

### Statistical analysis

The significant differences between three groups were analyzed using the Kruskal—Wallis test. And also Post hoc test procedures were performed using the Tukey test. A p-value of 0.05 was considered significant. All statistical analyses were performed using IBM SPSS version 22 software.

## Results

The results of the recovery test for the three preliminary operations for ICP-OES analysis are shown in Table 1. As indicated there, the dilution of samples was done in three ways; direct dilution with super pure HNO_3_+60%HClO_4_, direct dilution with super pure HNO_3_ and direct dilution with ultrapure distilled water. Recoveries after direct dilution with super pure HNO_3_ ranged from 81% to 121%, and hence this method was selected and was used for all further analyses.

**Table 1:** Recovery tests for the preliminary operations for ICP-OES analysis in saliva.

	Direct dilution with super pure HNO_3_+60%HClO_4_		Direct dilution with super pure HNO_3_		Direct dilution with Distilled ultrapure water	
**Spike concentration (μgl** ^**-1**^ **)**	5	10	5	10	5	10
**Mn**	104	114	110	115	107.8	81
**Cu**	145	129	91	96	125	106
**Zn**	132	180	84	86	308	64
**Mg**	321	167	108	111	80	510
**Pb**	95	88	99	107	75	63
**Rb**	1852	867	108	112	N.D.	N.D.
**Ca**	121	102	121	119	99	114
**Cd**	88	80	81	79	95	91
**Mo**	65	52	87	85	20	15
**Sr**	187	171	111	107	117	103

N.D.: not detected

Daily variations in salivary concentrations and also salivary secretion rates of trace metals are shown in Table 2. The minimum and maximum concentrations for each element were measured for 7 days but mentioned for 2 days. We do not mention the average of variations for each element in all 7 days; because, in the final result, they have no significant influence and it is suggested for further researches not to repeat such a test because of being not economic. Unstimulated salivary secretion rate is obtained by multiplying the mean salivary concentration by the salivary flow rate [13]. In order to estimate the daily variation in the levels of trace elements in saliva, change in concentration or change in secretion rate were expressed as the percent ratio of the mean concentration or the secretion rate of the next day sample vs. the first day sample. As Table 2 shows, the concentration of each metal in saliva as well as salivary secretion rate changes widely from day to day.

**Table 2:** Daily changes in salivary concentrations and salivary secretion rates of trace elementary.

	(%) Change in secretion rate		(%) Change in metals concentration	
Metals	Minimum	Minimum	Minimum	Minimum
**Mn**	32.3	133.6	33.7	129.3
**Cu**	59.2	138.2	53.2	142.3
**Zn**	78.0	129.0	71.4	124.8
**Mg**	41.1	214.0	38.4	216.0
**Pb**	66.7	133.3	59.7	125.7
**Rb**	73.0	126.4	69.7	124.8
**Ca**	80.0	111.2	78.4	172.5
**Cd**	55.6	166.7	52.4	168.7
**Mo**	35.7	178.6	29.2	210.3
**Sr**	34.5	206.9	32.1	198.8

Age, salivary flow rate, the concentration of blood contamination and salivary metal concentrations in groups 1–3 are shown in Table 3. In Table 3, for the first and second group we have 15 different samples taken from 15 participants (N = 15) for 7 days. But for the third group, we have 15 different samples for 15 participants for 2 times in only one day. There were significant differences between the salivary flow rates (P˂0.05) in groups 1–3 and the results of previous studies finding a reduction in the amount of saliva in chemical warfare injures are re-confirmed [11]. In contrast, since transferrin values of all participants, both healthy subjects and chemical warfare injures were below 1.0 mg dl^-1^, and there is no significant difference between blood contaminations of saliva in the groups. As Table 3 indicates, only zinc and copper concentrations showed significant differences between the groups.

**Table 3:** Age, salivary flow rate, the level of blood contamination and salivary concentrations of trace metals.

		Healthy Subjects	Chemical warfare injures		
		Group 1	Group 2	Group 3	P-value
**Age (Year)**		45.74±2.1	46.5±2.4	46.16±1.8	**0.83**
**Flow rate (ml/min)**		0.65±0.1	0.37±0.2	0.48±0.3	**0.002** ^**a**^
**Blood contamination(mg dl** ^**-1**^ **)**		0.13±0.1	0.16±0.1	0.14±0.1	**0.22**
**Salivary Concentration (μgl** ^**-1**^ **)**	**LOD(μgl** ^**-1**^ **)**		`		
**Mn**	1.00	4.64±1.9	4.30±2.0	4.30±2.03	**0.92**
**Cu**	1.00	15.3± 5.4	45.7± 13.7	29± 8.6	**0.02** ^**a**^
**Zn**	1.00	37± 9.0	12.2± 3.6	20.6± 10.0	**0.01** ^**a**^
**Mg**	1.00	206.80±140.0	189.20±103.4	172.40±124.1	**0.827**
**Pb**	1.00	3.10±1.0	2.02±0.7	2.01±1.0	**0.169**
**Rb**	1.00	71.20±15.0	67.00±15.2	58.80±14.8	**0.56**
**Ca**	0.01	22484.6± 2776.0	19417±2916.0	22033± 2532.0	**0.125**
**Cd**	0.10	0.180±0.1	0.160±0.1	0.164±0.1	**0.879**
**Mo**	0.10	0.280±0.2	0.200±0.1	0.220±0.1	**0.727**
**Sr**	1.00	2.900±2.2	2.200±1.8	2.300±1.1	**0.76**

- Data are expressed as the mean±S.E; n = 15 in each group. Group 1, Healthy subjects; Group 2, 3; Chemical warfare injures.

- Limit of detection (LOD) for each element was calculated as three times the standard deviation of the Healthy subjects samples.

- Kruskal—Wallis test statistics for comparison,

^a^P < 0.05

The concentration of Cu in saliva is significantly increased in chemical warfare injures with respect to the healthy subjects as follows: healthy subjects 15.3± 5.45 (p.p.b), patients (group 2) 45.77±13.65 and patients (group 3) 29 ±8.51 (P <0.02). In contrast, zinc is significantly decreased in the patients as follows: healthy subjects 37 ± 9.03, (p.p.b) patients (group 2) 12.2 ± 3.56 and the patients (group 3) 20.6 ±10.01 (P < 0.01). It is important to note that all metals concentrations are higher, except Rb, in the second group than the third group. The salivary secretion rates of trace elements in groups 1–3 are presented in Table 4.

**Table 4:** Salivary secretion rates (ng min^-1^) of trace metals in healthy subjects (group 1) and chemical warfare injures (group 2 and group 3).

Metal	Healthy subjects (Group1)	Chemical warfare injure (Group 2)	Chemical warfare injure (Group 3)	P-value
**Mn**	3.16±1.5	1.68±0.6	2.03±0.9	**0.041** ^**a**^
**Cu**	10.03±1.7	17.28±6.1	13.74±6.2	**0.010** ^**b**^
**Zn**	24.27±4.0	4.68±1.7	9.76±4.4	**0.001** ^**b**^
**Mg**	125.17±58.8	72.65±25.7	81.71.10±36.6	**0.025** ^**a**^
**Pb**	2.04±0.9	0.77±0.3	0.95±0.9	**0.014** ^**a**^
**Rb**	45.6±5.7	25.72±9.1	27.87±12.5	**0.027** ^**a**^
**Ca**	14717.14±3039.7	7456.12±2636.0	10443.64±4675.5	**0.032** ^**a**^
**Cd**	0.12±0.1	0.06±0.1	0.075±0.1	**0.029** ^**a**^
**Mo**	0.177±0.1	0.076±0.1	0.104±0.1	**0.018** ^**a**^
**Sr**	1.854±1.4	0.812±0.1	1.148±0.6	**0.049** ^**a**^

- Data are expressed as the mean±S.E; n = 15 in each group. Group 1, Healthy subjects; Group 2, 3; Chemical warfare injures.

- The Salivary secretion rate was computed by multiplying the mean salivary concentration by the salivary flow rate.

^a^ P<0.05

^b^ P<0.01

The salivary secretion rates of all trace metals indicated significant differences between healthy subjects and chemical warfare injured groups. Although, there are no significant differences between salivary secretion rates of trace metals in two and three groups, in both groups the biggest changes is related to Zn and Cu. In this experimental task, we did not find any significant differences in the concentrations of all elements (especially copper) for those healthy subjects that have different numbers of teeth restored (data not shown). All elements had no meaningful difference with the number of metal restorations.

## Discussion

Due to the rapid development of analytical research instrumentation, there is more accurate means of measuring concentration of trace elements in saliva. ICP-OES device is widely accepted and used as one of the best tools in this field. In this experiment, the limit of detection (LOD) of each metal was ranged from 0.01 to 1 μgl^-1^, and no interference in the multi-metals analysis was observed- highlighting the sensitivity and the usefulness of ICP-OES. Among the three methods tested, the direct dilution with super pure HNO_3_ indicated the most stable recovery range. However, others have used a mixture of HClO_4_ and HNO_3_ to measure a limited number of trace metals concentration such as Mn and Cu [14]. The method used in this investigation for the simultaneous measurement of a large number of trace metals is more accurate than other methods. Direct dilution with ultrapure water is not a suitable method; because, it may not totally remove large and small organic compounds from the samples. Consequently, using super pure HNO_3_ is needed to remove impurities of saliva samples and to control sample contamination. When we collected saliva samples from healthy subjects in two different days, we found many changes in salivary levels of trace metals. We concluded from these results that the salivary concentration and salivary secretion rates of trace elements of each individual depend on his nutritional and hormonal status. In order to control these limitations, we examined salivary flow rate, salivary secretion rate and blood pollution in saliva samples, simultaneously. Because blood contamination of saliva samples can artificially alter the concentration of trace metals, the amount of blood contamination in saliva samples must first be measured [15], which was done through measuring of transferrin concentrations in saliva samples. Generally, if the concentration of transferrin in saliva is more than 1.0 mg dl^-1^, blood contamination in saliva is positive and if it is less than 1.0 mg dl^-1^, the test is nullified. Our results show that transferrin concentration in the saliva samples of all participants were below 1.0 mg dl^-1^; so, blood contamination in saliva samples was minimal. Furthermore, our results indicate that there is no significant difference between metal concentrations in saliva samples of healthy subjects which shows that the number of their restored teeth doesn't affect the metal concentrations. This is consistent with previous report that showed no significant difference in the metals concentrations between groups in which all carious teeth were treated [14, 16, 17]. Koji Watanabe and et al showed that the Cu levels in patients with untreated carious teeth were meaningfully higher than those healthy subjects, and they are increased along with the number of untreated teeth [14]. Although, the participants in this investigation have a different number of missing or restored teeth, there are almost no carious teeth. In this study, salivary concentrations of trace elements obtained for normal subjects were different from those obtained in other studies [14, 17,18 19], which are attributed to the differences in samples preparation, devices used, the food variation, procedures, blood or environmental pollution and exposure to metals. It should be noted that the participants in this study live in an area where there are very few factories and environmental pollution. Accordingly, the trace metals concentrations of saliva samples in healthy subjects are much lower than the values in previous research.

The most important purpose of this experimental task is to consider the influence of mustard gas on the salivary concentration and salivary secretion rates of Mn, Cu, Zn, Mg, Pb, Rb, Ca, Cd, Mo and Sr in the chemical warfare injures. To the best of our knowledge, there has not been any reported similar study in this area and the present study is the first. In a study on 236 Iranian chemical warfare injures, the maximum effects within 2–28 months after exposure to mustard gas were reported as the following: 78% in the respiratory system, 31% in skin, and 36% in eyes [20]. In another study on 40 chemical warfare injures, 16–20 years after exposure to mustard gas, it was found that the most common complication is related with chronic respiratory disease [21]. Almost all of the chemical warfare injures subjected to this study are suffering from respiratory diseases. In this case, Cu and Zn levels in salivary concentration and salivary secretion rates are significantly increased and decreased for the two metals, respectively. Such changes in chemical war veterans who did not use regular medicine (group 2), were higher than chemical war veterans who used medicine. Subsequently, we conclude that the use of the Salbutamol spray has a positive influence on salivary concentration, salivary flow rate and salivary secretion rate. Salbutamol (INN) or albuterol (USAN) with formula: C_13_H_21_NO_3_, is a short-acting β2-adrenergic receptor agonist used for the release of bronchospasm in conditions such as asthma and chronic obstructive pulmonary diseases [22]. In various diseases such as anemia, Wilson's disease, leukemia and lymphoma, acute and chronic infection, biliary cirrhosis, carious teeth, hemochromatosis, collagen diseases, hypothyroidism, hyperthyroidism, etc., the copper concentration is increased and mainly associated with increased C-reactive protein (CRP) [14, 23–25]. Several diseases such as HIV, acute infection, burns, diabetes, a condition that causes albumin decrease, taste disorder, nephritic syndrome, inflammatory diseases, malabsorption, Crohn's disease, myocardial infarction, etc., the concentration of zinc is reduced [18, 23–26]. A significant reduction in Zn concentration in the saliva of chemically injured patients may be due to pulmonary inflammation because use of the Salbutamol spray gives patients relief although does not cure the inflammation.

## Conclusions

The main purpose of this research is to consider the influence of mustard gas on the salivary concentration of trace metal and salivary secretion rate in the chemical warfare injures. We found that the optimum ICP-OES results were obtained when the saliva samples were diluted with super pure HNO3. Trace elementary concentration in saliva showed daily variations. Saliva concentrations of trace metals were not depended upon the number of teeth restored. The results obtained for saliva concentration of trace metals due to lack of blood contamination and environmental interference were much lower. Notably, the reduction of Zn and the increase of Cu may be used as a marker for diagnosing exposure of people to mustard gas, provided that the findings of this study are reproduced in a larger cohort of subjects.
